# Opposing prognostic roles of tumor-associated and circulating MMP8 in colorectal cancer

**DOI:** 10.1007/s10238-026-02131-5

**Published:** 2026-04-03

**Authors:** Iivari Loukasmäki, Anne Tuomisto, Päivi Sirniö, Meeri Kastinen, Henna Karjalainen, Ville K. Äijälä, Vilja V. Tapiainen, Taina Tervahartiala, Timo Sorsa, Vesa-Matti Pohjanen, Taneli T. Mattila, Outi Lindgren, Jukka Rintala, Sanna Meriläinen, Juha Saarnio, Tero Rautio, Markus J. Mäkinen, Juha P. Väyrynen

**Affiliations:** 1 Translational Medicine Research Unit, Medical Research Center Oulu, Oulu University Hospital, University of Oulu, Aapistie 5A, 90220 Oulu, Finland; 2 Department of Oral and Maxillofacial Diseases, Institute of Dentistry, Helsinki University Central Hospital, Helsinki, Finland; 3 Department of Oral Diseases, Karolinska Institutet, Huddinge, Sweden

**Keywords:** Colorectal cancer, MMP8, Immune cells, Prognosis

## Abstract

Local and systemic inflammation play key roles in colorectal cancer (CRC). Matrix metalloproteinase 8 (MMP8), an enzyme involved in extracellular matrix remodeling, has been linked to both inflammatory processes and cancer progression. This study investigates MMP8 serum concentration and MMP8 expression within the tumor microenvironment, and their relationship with tumor characteristics, inflammation, and survival. The study included 776 stage I-IV CRC patients. MMP8^+^ cell densities within tumors were analyzed using immunohistochemistry combined with digital image analysis, while preoperative serum MMP8 levels were analyzed using an immunofluorometric assay. Survival analyses were performed using the Kaplan-Meier method and Cox regression models. Correlations with systemic inflammatory markers and immune cell densities were assessed using linear regression models. We found that MMP8 was predominantly expressed by CEACAM8^+^ granulocytes. High MMP8^+^ cell density within tumors was associated with favorable prognostic factors, including low disease stage, absent lymphovascular invasion, and mismatch repair (MMR) deficiency, but also with improved cancer-specific survival independent of these factors. The multivariable HR for low (vs. high) MMP8^+^ cell density was 1.76 (95%CI 1.18–2.60). MMP8^+^ cell density positively correlated with T-cell densities, but not with serum MMP8 levels or conventional systemic inflammation markers. In contrast, high serum MMP8 levels were associated with systemic inflammation and worse survival, but did not remain an independent prognostic factor in multivariable models. In conclusion, serum MMP8 levels do not correlate with MMP8 expression in the tumor microenvironment, suggesting distinct roles for circulating and tumor-associated MMP8 in CRC progression. High MMP8^+^ cell densities within tumors are associated with better prognosis and local immune activity, while elevated serum MMP8 levels indicate systemic inflammation and advanced disease.

**Supplementary Information:**

The online version contains supplementary material available at 10.1007/s10238-026-02131-5.

## Introduction

Colorectal cancer (CRC) is the third most common type of cancer, and it causes the second most cancer deaths in Europe [1]. In 2022, approximately 1.93 million new CRC cases were diagnosed globally and 0.90 million died of the disease [2]. The incidence of CRC is still growing and has been estimated to reach 3.2 million new cases a year in 2040 [3]. CRC tumors trigger a local inflammatory response [4], and a dense inflammatory cell infiltrate is usually associated with good prognosis. However, tumor-associated inflammatory cells can also facilitate tumor progression, invasion, and metastasis [5, 6]. Especially, innate immune cells may promote tumor progression by secreting cytokines, chemokines, and proteases [6]. In addition, CRC can induce systemic inflammation, with tumor cells and local inflammatory cells releasing cytokines which end up in circulation and stimulate acute phase protein production in the liver [7]. This systemic inflammation, observed in 21–41% of CRC patients, is associated with advanced disease, tumor progression, metabolic alterations, and poor survival [8].

Matrix metalloproteinases (MMPs) are proteolytic enzymes primarily known for degrading extracellular matrix components [9]. MMP8, also called neutrophil collagenase, is an endopeptidase that cleaves collagens I, II, and III and participates in embryogenesis, cell proliferation, tissue remodeling, and the pathogenesis of various diseases, including cancer [10]. MMP8 is mainly secreted by neutrophils. Beyond collagen degradation, MMP8 cleaves certain adhesion proteins, growth factors, insulin receptors and cytokines [11, 12]. Previous studies have linked high serum MMP8 concentrations with poor CRC prognosis and high serum concentrations of inflammatory markers, such as CRP [13]. MMP8 might therefore contribute to systemic inflammation in CRC by cleaving cytokines potentially reflecting dysregulated immune responses [14], and has been proposed as a potential immune-related biomarker for CRC [15].

With this background, we aimed to investigate whether serum MMP8 concentration and MMP8 expression within the tumor microenvironment could predict patient survival in a large CRC cohort [12]. The hypothesis was that high serum MMP8 concentration would be associated with strong MMP8 expression in tumor tissue and with worse prognosis.

## Methods

### Study population

**Fig. 1:**
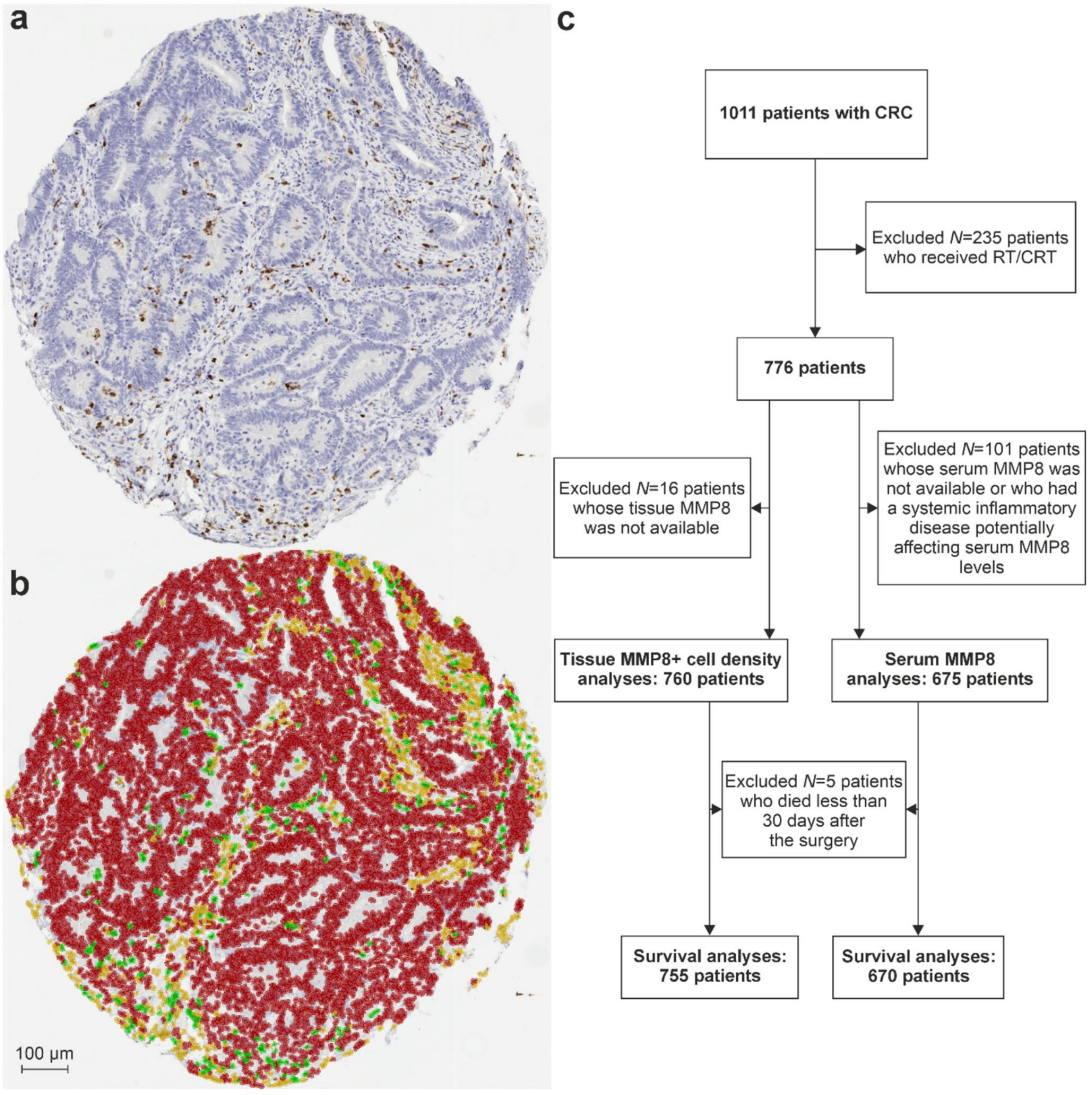
MMP8 immunohistochemistry and a flowchart of the patients analyzed in the study. (**A**) A representative example of MMP8 immunohistochemistry in colorectal cancer. (**B**) Cell detection and classification using QuPath. In the image analysis result image, red color represents cancer cells, green MMP8^+^ cells and yellow other cells. (**C**) A flowchart of the patients analyzed in the study with exclusion criteria. Abbreviations: CRC, colorectal cancer; RT/CRT, radiotherapy or chemoradiotherapy

The study included CRC patients who underwent surgery at Oulu University Hospital in 2006–2020 and provided informed consent (*N* = 1,011) [16]. Preoperative serum samples and resected tissues were analyzed. MMP8 serum levels for 271 of these patients operated on between 2006 and 2014 had been analyzed in a previous study [13], but tissue MMP8 expression had not been assessed before. Patients who had received preoperative chemotherapy or radiotherapy (*N* = 235) were excluded, leaving 776 patients for analysis. Of these, 760 of them had available tissue MMP8 data, and 675 had usable serum MMP8 data after excluding patients with missing data or conditions potentially affecting serum MMP8 levels, such as chronic inflammatory conditions including rheumatoid arthritis, chronic obstructive pulmonary disease, or asthma (*N* = 101). For survival analyses, patients who died within 30 days of surgery (*N* = 5) were additionally excluded as probable surgery-related deaths. A flowchart detailing patient selection is shown in Fig. 1C.

Clinical data were retrieved from patient records, while follow-up information was obtained from clinical records and Statistics Finland. Clinical details, such as the American Association of Anesthesiologists (ASA) classification (assessing overall disease burden and anesthesia fitness), were collected as part of routine care and retrieved from medical records. Follow-up extended until December 31, 2021, with a median duration of 5.6 years for censored cases (IQR 3.7–9.3). Up to 10 years of follow-up data were utilized, as most CRC-related deaths occur within this timeframe [17]. The clinical endpoints were cancer-specific survival (CSS), defined as the interval from surgery to death due to CRC, and overall survival (OS), defined as the interval from surgery to death from any cause. The reporting recommendations for tumor marker prognostic studies (REMARK) were considered in the study design [18].

### Serum samples and analyses

Preoperative serum samples were collected 0–30 days before surgery and stored at −70˚C. Blood neutrophil and lymphocyte counts, as well as serum concentrations of C-reactive protein (CRP) and albumin, were measured in the laboratory of Oulu University Hospital, with analyses performed for 730, 730, 722, and 766 samples, respectively [19]. The modified Glasgow Prognostic Score (mGPS) was determined using CRP and albumin levels. A score of 2 was assigned to patients with elevated CRP (> 10 mg/L) and hypoalbuminemia (< 35 g/L). A score of 1 was given to those with elevated CRP but normal albumin levels, while a score of 0 was assigned to patients with normal levels of both CRP and albumin. Serum MMP8 levels were quantified using a time-resolved immunofluorometric assay (IFMA) that detects active MMP8 [15]. These analyses had previously been conducted for 271 patients operated on between 2006 and 2014 [13], and were now extended to include patients operated on until 2020. Serum IL6 analyses for cases from 2010 to 2020 were performed by Olink Target 96 Immuno-Oncology panel [20], yielding successful results for 603 cases.

### Histopathological analyses

Tumor specimens were fixed in 10% formalin, then embedded in paraffin, and analyzed using hematoxylin & eosin staining. Tumor TNM stage was determined by the UICC/AJCC (Union for International Cancer Control/The American Joint Committee on Cancer) criteria and tumor grade according to the WHO 2019 criteria. Lymphovascular invasion was recognized as tumor cells within vascular spaces and tumor necrosis percentage was evaluated according to the established criteria [16].

### Immunohistochemistry and image analysis

Tissue microarrays (TMA) were constructed using 1 mm diameter cylindrical cores, with four cores per tumor sampled (two from the central region and two from the invasive front). The TMA sections were stained using the BOND RX staining system (Leica) with the BOND Polymer Refine Detection kit (Leica, DS9800), a rabbit monoclonal MMP8 antibody (clone E1C4L, Cell Signaling Technology, dilution 1:100), and antigen retrieval with BOND Epitope Retrieval Solution 2 (Leica, AR9640). Additionally, a double immunohistochemistry assay was employed to detect MMP8 along with CEACAM8 (CD66b), a marker for the granulocytic cell lineage. This assay was also performed using Leica BOND RX. The first staining cycle utilized BOND Polymer Refine Red Detection kit (Leica, DS9390), CEACAM8 (CD66b) antibody (clone G10F5, Biolegend, dilution 1:100), and antigen retrieval using BOND Epitope Retrieval Solution 2 (Leica, AR9640), and the second cycle was performed with the BOND Polymer Refine Detection kit (Leica, DS9800), a rabbit monoclonal MMP8 antibody (clone E1C4L, Cell Signaling Technology, dilution 1:200), and BOND Epitope Retrieval Solution 1 (Leica AR9961).

The expression of the MMP8 protein in the tumor tissue was analyzed using machine learning-based methods integrated into the open-source QuPath digital microscopy software [21, 22]. TMA cores were annotated using the *TMA dearrayer* function. A *pixel classifier* was trained using the random trees algorithm to detect representative tissue for analysis, while excluding white space, mucin, necrosis, and folds. Cell detection was followed by the calculation of additional Haralick’s texture features (*Add intensity features*) and smoothed features (*Add smoothed features*). A random trees *object classifier* was then trained to detect cells expressing MMP8, as well as MMP8^−^ tumor cells and other cells. The training was performed by manually annotating representative cells on 90 tissue microarray (TMA) cores across the dataset selected to capture the range of staining intensity and common artifacts present in the dataset. The performance of the classifier was assessed during the training by visually evaluating the correspondence of cell classification and Diaminobenzidine staining. For quantitative validation, we compared the predictions of the classifier with manual single-cell annotations (*N* = 111,954 cells across 20 TMA cores not used in classifier training). For the distinction between MMP8^+^ and MMP8^−^ cells, the classifier achieved an accuracy of 0.983, precision of 0.679, recall (sensitivity) of 0.831, F1 score of 0.747, and specificity of 0.988. These metrics indicate high overall correctness and excellent specificity, with good sensitivity and F1 score for detection of MMP8^+^ cells. As the study endpoint is MMP8⁺ cell density per core, we assessed correlation between automated and manual MMP8⁺ cell counts across the 20 validation cores. Automated and manual counts were strongly correlated (Spearman rho = 0.97), supporting the robustness of the density quantification despite imperfect per-cell precision.

Example images of MMP8 immunohistochemistry and cell classification can be seen in Fig. 1A-B. The data from QuPath were further processed using RStudio and R statistical programming (v. 4.3.1) to quantify the overall densities of MMP8^+^ cells of each tumor. All MMP8 analyses were performed blinded to the clinical outcomes.

Additionally, CD3^+^ and CD8^+^ T cell densities within tumors were calculated from TMA cores, as previously described [23]. *BRAF* V600E mutation and mismatch repair (MMR) statuses of the tumors were defined from the TMA cores by immunohistochemistry [16].

### Statistical analyses

Statistical analyses were conducted using IBM SPSS Statistics 29 software (IBM Corporation, Armonk, NY, USA). A *P*-value of < 0.05 was considered statistically significant. Associations between serum MMP8 levels and categorical variables were analyzed using the Mann-Whitney test for comparisons with two categories, and the Kruskal-Wallis test for variables with three or more categories. The associations between two categorical variables were assessed using the Chi-square test or Fisher’s exact test. Pearson’s or Spearman’s correlation tests were employed to explore the correlations of MMP8^+^ cell densities and serum MMP8 levels with T cell densities, tumor necrosis percentage, blood cell counts and serum inflammatory markers. The continuous variables with positive skewness were normalized using logarithmic transformation. Linear regression models were used to adjust the correlation coefficients for the following covariates: age (< 65, ≥ 65), sex (male, female), tumor localization (colon, rectum), T class (T1-T2, T3-T4), N class (N0, N1-N2), M class (M0, M1), BRAF status (wild-type, mutant), MMR status (proficient, deficient), and tumor grade (low, high). Model assumptions, including linearity, normality, and homoscedasticity, were verified through residuals’ normal probability plots and scatterplots comparing residuals to predicted values.

Receiver operating characteristic (ROC) analysis was performed to evaluate the ability of serum MMP8 levels and MMP8^+^ cell densities to discriminate 10-year CSS and OS. Discrimination was quantified using the area under the curve (AUC) [24]. Optimal cut-off points were determined by maximizing Youden’s index for CSS. For MMP8^+^ cell density, this yielded the cut-off of 220 cells/mm^2^. For serum MMP8, we used a pre-specified cut-off of 100 ng/mL, adopted from prior work [13], and additionally performed a sensitivity analysis deriving a cohort-specific serum MMP8 cut-off (56.6 ng/mL) using ROC analysis and Youden’s index.

Categorical variables of serum MMP8 levels and MMP8^+^ cell densities in relation to CSS and OS were examined using Kaplan-Meier curves and Cox regression models. Multivariable Cox proportional hazards regression models were adjusted for sex, age (< 65, 65–75, > 75), year of operation (2006–2010, 2011–2015, 2016–2020), tumor location (proximal colon, distal colon, rectum), disease stage (I–II, III, IV), tumor grade (low-grade, high-grade), lymphovascular invasion (negative, positive), mismatch repair (MMR) status (proficient, deficient), *BRAF* status (wild-type, mutant).

## Results

### MMP8 is expressed by granulocytes in the tumor microenvironment

MMP8 immunohistochemistry was successfully evaluated for 760 colorectal cancer patients and serum MMP8 data was available for 675 patients. MMP8 positivity was mainly detected in cells morphologically resembling neutrophils. To confirm the association between MMP8 expression and granulocyte lineage, CEACAM8-MMP8 double immunohistochemistry was performed on a TMA slide comprising 25 tumors, of which 24 contained granulocytes (Figure S1). Among these, an average of 95% (SD 11%) of CEACAM8^+^ granulocytes were MMP8^+^, while an average of 91% (SD 14%) of MMP8^+^ cells were CEACAM8^+^ granulocytes. Thus, an average of 9% of MMP8^+^ cells were not CEACAM8^+^. Morphologically, these CEACAM8^−^MMP8^+^ cells were most often either mononuclear/fibroblast-like stromal cells (42%) or polymorphonuclear leukocytes without clear CEACAM8 staining (58%). We did not observe convincing MMP8 expression in epithelial tumor cells or endothelial cells in these double-stained sections.

### MMP8^+^ cell densities within tumors and serum MMP8 exhibit distinct prognostic roles

**Fig. 2:**
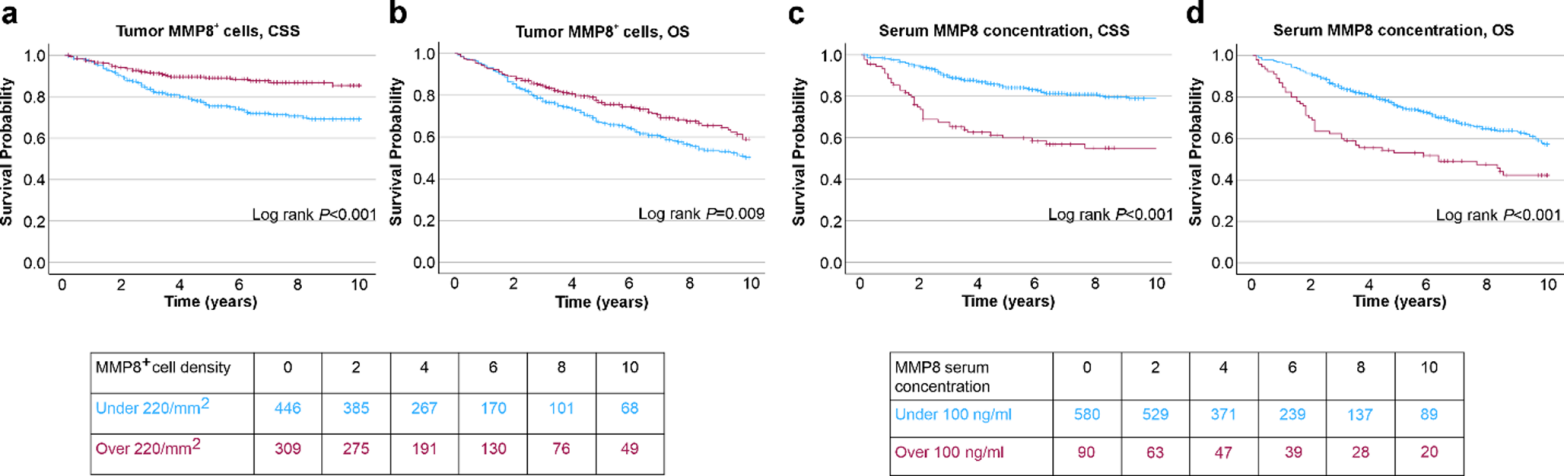
Kaplan-Meier survival analyses. The associations of tumor MMP8^+^ cell density with (**A**) cancer-specific survival (CSS) and (**B**) overall survival (OS) and the associations of serum MMP8 concentration with (**C**) CSS and (D) OS

As our main aim, we analyzed the prognostic significance of the tumor-associated MMP8^+^ cell density and serum MMP8 levels. There were 258 deaths, including 144 cancer deaths, in the 10-year follow-up period among patients with tumor MMP8^+^ cell density data and 223 deaths, including 129 cancer deaths among patients with serum MMP8 data. ROC analysis demonstrated a moderate capacity of both MMP8^+^ cell densities (AUC = 0.625 for CSS and 0.576 for OS) and serum MMP8 levels (AUC = 0.626 for CSS and 0.617 for OS) to discriminate survivors from non-survivors (Figure S2) and identified an optimal cut-off value of 220/mm^2^ for MMP8^+^ cell densities in survival analyses. Kaplan-Meier survival functions showed higher CSS and OS probability for patients with higher MMP8^+^ cell densities within tumors (CSS log rank *P* < 0.001, OS log rank *P* = 0.009) (Fig. 2). Multivariable Cox regression confirmed that lower MMP8^+^ cell densities were associated with shorter CSS (HR 1.76, 95%CI 1.18–2.60) (Table 1).

**Table 1:** Univariable and multivariable Cox regression models for cancer-specific survival and overall survival according to MMP8^+^ cell density in tumor tissue and MMP8 concentration in serum

	No. of cases	Colorectal cancer-specific survival			Overall survival		
		No. of events	Univariable HR (95% CI)	Multivariable HR (95% CI)	No. of events	Univariable HR (95% CI)	Multivariable HR (95% CI)
Tumor MMP8^+^ cell density							
≤220 cells/mm^2^	446	109	2.24 (1.53–3.28)	1.76 (1.18–2.60)	170	1.40 (1.08–1.81)	1.19 (0.92–1.56)
>220 cells/mm^2^	309	35	1 (referent)	1 (referent)	88	1 (referent)	1 (referent)
* P*			< 0.001	0.005		0.010	0.189
Serum MMP8 concentration							
≤100 ng/mL	580	92	1 (referent)	1 (referent)	174	1 (referent)	1 (referent)
>100 ng/mL	90	37	2.98 (2.03–4.37)	1.28 (0.83–1.95)	49	1.98 (1.44–2.72)	1.09 (0.77–1.54)
* P*			< 0.001	0.261		< 0.001	0.637

Abbreviations: CI, confidence interval; HR, hazard ratio. Multivariable Cox proportional hazards regression models were adjusted for sex, age (< 65, 65–75, > 75), year of operation (2006–2010, 2011–2015, 2016–2020), tumor location (proximal colon, distal colon, rectum), disease stage (I–II, III, IV), tumor grade (well/moderately differentiated, poorly differentiated), lymphovascular invasion (negative, positive), mismatch repair (MMR) status (proficient, deficient), *BRAF* status (wild-type, mutant). The missing data in the serum MMP8 model (*n* = 6 for *BRAF* status) were included in the majority category (*BRAF* wild-type) to limit the degrees of freedom

Conversely, Kaplan-Meier curves showed that higher serum MMP8 concentrations (pre-specified cut-off adopted from prior research: 100 ng/mL) were associated with shorter CSS and OS (log rank *P* < 0.001 for both) (Fig. 2). Serum MMP8 also represented an adverse prognostic factor for CSS (HR for high vs. low 2.98, 95%CI 2.03–4.37) and OS (HR for high vs. low 1.98, 95%CI 1.44–2.72) in univariable Cox regression models, but did not remain an independent prognostic factor in multivariable models (CSS: HR for high vs. low 1.28, 95%CI 0.83–1.95; OS: HR for high vs. low 1.09, 95%CI 0.77–1.54) (Table 1). Full multivariable Cox regression models are displayed in Table S1. Similar results were obtained when applying a lower serum MMP8 cut-off value (56.6 ng/mL). Serum MMP8 was an adverse prognostic factor for both CSS and OS in univariable Cox regression models but again did not remain independent in multivariable analyses (Table S2).

### Association of MMP8^+^ cell density with clinicopathological features

The relationships between MMP8^+^ cell densities (as a continuous variable) and patient characteristics were analyzed using Mann-Whitney test or Kruskal-Wallis test, and the results are summarized in Table 2. The strongest associations were observed with tumor localization at proximal colon or rectum (*P* = 0.002), lower tumor invasion depth (*P* < 0.001), fewer nodal metastases (*P* < 0.001), absence of distant metastases (*P* = 0.006), lower TNM stage (*P* < 0.001), absence of lymphovascular invasion (*P* < 0.001), presence of *BRAF* mutation (*P* < 0.001), and MMR deficiency (*P* < 0.001). When dichotomized using the prognostically informative cut-off (220 cells/mm²), high intratumoral MMP8⁺ cell density was associated with tumor location (*P* = 0.013), lower tumor invasion depth (*P* < 0.001), fewer nodal metastases (*P* < 0.001), absence of distant metastases (*P* = 0.009), lower TNM stage (*P* < 0.001), absence of lymphovascular invasion (*P* = 0.001), presence of *BRAF* mutation (*P* = 0.020), and MMR deficiency (*P* < 0.001) (Table S3).

**Table 2:** Baseline patient characteristics according to tumor MMP8^+^ cell densities and serum MM8 concentrations

Group	*N*	Tumor MMP8^+^ cells (1/mm^2^), median (IQR)	*P*	*N*	Serum MMP8 (ng/mL), median (IQR)	*P*
All cases	760	162.9 (65.5–365.7)		675	32.8 (18.8–67.2)	
Age						
< 65 ≥ 65	229 531	177.1 (59.2–350.6) 159.8 (66.6–368.6)	0.951	210 465	33.5 (18.4–63.6) 32.4 (19.2–70.2)	0.687
Sex						
Male Female	402 358	156.1 (66.9–353.2) 168.7 (62.5–391.5)	0.624	366 309	31.9 (18.8–63.7) 34.0 (18.8–72.5)	0.324
Tumor location						
Proximal colon Distal colon Rectum	319 204 237	174.7 (76.8–441.7) 129.2 (50.0–285.6) 177.1 (67.5–399.0)	0.002	272 183 220	32.7 (18.8–70.4) 33.8 (20.0–67.4) 32.0 (18.0–64.2)	0.569
ASA						
1 2 3 4	39 289 313 61	134.4 (58.3–269.9) 172.6 (71.4–346.8) 167.1 (58.5–396.9) 156.4 (86.7–351.9)	0.918	36 259 272 50	23.4 (14.6–36.3) 32.4 (18.4–64.0) 31.4 (18.0–65.2) 37.5 (27.6–72.6)	0.034
Depth of invasion						
T1 T2 T3 T4	38 177 434 111	312.1 (108.4–689.9) 231.1 (91.7–419.8) 137.8 (58.3–310.8) 133.0 (46.1–293.5)	< 0.001	47 154 384 90	28.0 (15.6–47.2) 30.2 (17.1–52.5) 36.0 (19.6–76.0) 33.4 (21.5–85.0)	0.002
Nodal metastases						
N0 N1 N2	440 192 128	193.2 (74.7–406.8) 131.5 (52.8–273.5) 136.5 (46.8–306.8)	< 0.001	390 174 111	32.0 (18.4–62.4) 36.6 (19.1–80.5) 36.4 (20.8–80.8)	0.103
Distant metastases						
M0 M1	676 84	169.9 (69.9–375.8) 108.6 (40.5–251.6)	0.006	599 76	31.4 (18.1–62.6) 62.3 (31.8–205.9)	< 0.001
TNM stage						
Stage I Stage II Stage III Stage IV	176 250 250 84	269.0 (102.9–484.8) 161.7 (67.1–364.3) 132.2 (54.0–283.6) 108.6 (40.5–251.6)	< 0.001	163 214 222 76	29.6 (17.2–48.8) 33.2 (19.4–71.8) 30.4 (17.6–67.2) 62.3 (31.8–205.9)	< 0.001
WHO grade						
Low High	650 110	157.9 (62.4–353.7) 191.9 (68.5–462.0)	0.068	579 96	32.2 (18.4–64.3) 42.3 (20.0–95.1)	0.034
Lymphovascular invasion						
No Yes	415 345	194.1 (85.1–412.6) 129.92 (48.3–295.1)	< 0.001	376 299	31.5 (18.8–61.3) 36.0 (18.8–77.6)	0.037
*BRAF* status						
Wild-type Mutant	653 107	155.1 (59.2–336.4) 235.5 (105.8–610.6)	< 0.001	581 88	32.2 (18.6–65.5) 37.7 (20.0–83.8)	0.228
MMR status						
MMR-proficient MMR-deficient	638 122	136.3 (56.0–311.2) 328.6 (160.5–580.0)	< 0.001	105 570	32.0 (18.4–65.1) 37.2 (20.0–80.4)	0.105
mGPS						
mGPS0 mGPS1 mGPS2	555 96 55	162.7 (65.4–351.0) 176.7 (71.3–382.5) 183.9 (76.8–488.6)	0.792	527 92 46	29.6 (17.2–59.2) 56.9 (31.6–116.7) 57.2 (27.9–149.3)	< 0.001

Abbreviations: ASA, American Society of Aneshesiologists; mGPS, modified Glasgow prognostic score; IQR, interquartile range; MMR, mismatch repair. *P* values are for Mann-Whitney or Kruskal-Wallis test. Missing data for ASA class: 58 for both tumor and serum data, BRAF: 6 for serum data, mGPS: 54 for tumor and 10 for serum data

MMP8^+^ cell densities within tumors positively correlated with T-lymphocyte densities (Linear regression: *P* < 0.001 for both CD3^+^ and CD8^+^ cells, Table 3). They also inversely correlated with tumor necrosis percentage (Pearson *r*=−0.110, *P* = 0.002), although this association lost significance in a multivariable linear regression model (beta − 0.005, *P* = 0.897).

**Table 3:** Correlations between tumor MMP8^+^ cell density, serum MMP8 concentrations, histological features, and systemic inflammation markers

Variable	Tumor MMP8^+^ cell density					Serum MMP8 concentration				
	*N*	Unadjusted		Adjusted		*N*	Unadjusted		Adjusted	
		Pearson r	*P* value	Beta	*P* value		Pearson r	*P* value	Beta	*P* value
Systemic inflammatory markers										
Serum MMP8	660	−0.019	0.626	0.021	0.586					
Blood neutrophil count	715	−0.032	0.391	−0.024	0.519	664	0.462	<0.001	0.432	<0.001
Blood lymphocyte count	715	−0.007	0.846	0.000	0.999	664	0.015	0.703	0.011	0.774
NLR	715	−0.019	0.606	−0.018	0.625	664	0.348	<0.001	0.321	<0.001
Serum CRP	707	−0.002	0.968	0.023	0.547	675	0.381	<0.001	0.314	<0.001
Serum albumin	750	−0.012	0.738	−0.007	0.852	665	0.012	0.764	0.038	0.314
Serum IL6	592	−0.008	0.839	0.012	0.768	551	0.295	<0.001	0.268	<0.001
Tumor properties										
CD3^+^ T-cell density	759	0.323	<0.001	0.235	<0.001	659	−0.118	0.002	−0.046	0.259
CD8^+^ T cell density	759	0.288	<0.001	0.196	<0.001	659	−0.108	0.005	−0.067	0.103
Tumor necrosis percentage	760	−0.110	0.002	−0.005	0.897	675	0.190	<0.001	0.143	<0.001

Abbreviations: NLR, Neutrophil/Lymphocyte Ratio; CRP, C-reactive protein; IL, interleukin. The adjusted beta coefficients and P values were calculated with linear regression models that included age (<65, ≥65), sex (male, female), tumor localization (colon, rectum), T class (T1-T2, T3-T4), N class (N0, N1-N2), M class (M0, M1), *BRAF* status (wild-type, mutant), MMR status (proficient, deficient), and tumor grade (low, high). Continuous variables that were not normally distributed were logarithmically transformed. The missing data in the serum MMP8 model (*n*=6 for *BRAF* status) were included in the majority category (*BRAF* wild-type) to limit the degrees of freedom

### Serum MMP8 reflects systemic inflammation but not tumor-associated MMP8^+^ cell densities

Unexpectedly, no correlation was found between serum MMP8 levels and MMP8^+^ cell densities within tumors when analyzed as continuous variables (Pearson *r*=−0.019, multivariable linear regression beta = 0.021, *P* > 0.1 for both). Concordantly, when dichotomized using the same prognostically informative cutoffs applied in the main analyses, serum MMP8 status and tissue MMP8 status showed poor concordance (Table S4). Both measures were low in 340/660 (51.5%) cases and both were high in 33/660 (5.0%). Overall agreement was 56.5% and Cohen’s κ was − 0.02.

Unlike tumor-associated MMP8^+^ cells, high serum MMP8 concentrations (analyzed as a continuous variable) were associated with unfavorable prognostic factors, including high ASA class (*P* = 0.034), greater depth of tumor invasion (*P* = 0.002), presence of distant metastases (*P* < 0.001), advanced TNM stage (*P* < 0.001), high WHO grade (*P* < 0.001), presence of lymphovascular invasion (*P* = 0.037), and high mGPS (*P* < 0.001) (Table 2). When dichotomized using the same serum MMP8 threshold as in survival analyses, high serum MMP8 was associated with greater tumor invasion depth (*P* < 0.001), presence of nodal (*P* = 0.010) or distant metastases (*P* < 0.001), advanced TNM stage (*P* < 0.001), higher WHO grade (*P* = 0.015), lymphovascular invasion (*P* < 0.001), and elevated mGPS (*P* < 0.001) (Table S3).

Serum MMP8 concentrations (as a continuous variable) were also positively correlated with tumor necrosis percentage (beta = 0.143, *P* < 0.001) and systemic inflammatory markers, including blood neutrophil count and neutrophil to lymphocyte ratio (NLR), serum CRP levels, and serum IL6 levels (all *P* < 0.001), in multivariable linear regression models (Table 3). The strongest correlation was observed with blood neutrophil count (beta = 0.432).

## Discussion

In this study, we identified distinct, contrasting roles for serum and tissue MMP8 in CRC. High MMP8^+^ cell densities in the tumor microenvironment were associated with MMR deficiency, higher T cell densities, and better disease outcome. In contrast, elevated serum MMP8 levels reflected systemic inflammation and showed univariable associations with poorer outcomes. These findings highlight the multifaceted role of MMP8 in CRC progression.

High densities of the tumor MMP8^+^ cells were predominantly associated with favorable prognostic factors, such as low TNM stage, absent lymphovascular invasion, and MMR deficiency [25, 26], but also with *BRAF* mutations, typically linked to worse prognosis [27]. Given that *BRAF* mutations and MMR deficiency are hallmarks of the serrated pathway in colorectal carcinogenesis, these findings suggest that MMP8 may exert context-dependent effects influenced by tumor heterogeneity or specific molecular pathways [28]. The observed positive correlation between MMP8^+^ cell and T cell densities suggests a potential role for MMP8 in contributing to anti-tumor immune responses, affirming previous results [29]. Double immunohistochemical analysis confirmed that MMP8 expression was largely restricted to granulocytic cells, further supporting its involvement specifically in granulocyte-mediated immune mechanisms [30]. Importantly, the double-stained analysis also showed that a minority of MMP8^+^ cells were CEACAM8^−^ (mean 9%), indicating that tissue MMP8 immunoreactivity is not exclusively granulocytic. Morphologically, these CEACAM8^−^MMP8^+^ cells most often resembled mononuclear/fibroblast-like stromal cells or polymorphonuclear leukocytes with absent or equivocal CEACAM8 staining, while we did not observe convincing expression in epithelial tumor cells. Because our main cohort analyses used single-plex MMP8 immunohistochemistry with automated quantification on TMAs, we cannot reliably separate granulocytic from non-granulocytic MMP8^+^ cells across the full cohort. Furthermore, the double-immunohistochemistry subset is limited (*n* = 25) and the CEACAM8^−^MMP8^+^ population is rare, precluding a reliable prognostic analysis in the present study. Larger studies using multiplex spatial methods are needed to determine whether the non-granulocytic MMP8^+^ compartment has independent prognostic relevance.

In contrast to tissue MMP8, high serum MMP8 concentrations were associated with adverse prognostic factors, including high ASA class, high TNM stage, high WHO grade, lymphovascular invasion, and increased mGPS [31, 32]. These findings are consistent with prior research linking elevated serum MMP8 levels to systemic inflammation and advanced disease [13, 33]. Serum MMP8 positively correlated with systemic inflammatory markers, such as NLR and serum CRP and IL6 concentrations, further supporting its role as a participant in systemic inflammatory responses [34]. Furthermore, serum MMP8 levels positively correlated with tumor necrosis, a hallmark of aggressive tumor biology and a potential contributor to systemic inflammation [20, 35]. These observations suggest that systemic MMP8 may contribute to a pro-inflammatory, pro-tumorigenic milieu, distinct from the localized effects of tumor-infiltrating MMP8^+^ cells. However, despite its associations with increased mortality in univariable analyses, serum MMP8 did not add substantial independent prognostic value beyond conventional clinicopathological markers in this cohort. Serum MMP8 may still be clinically informative as a complementary marker within multi-marker inflammatory risk models, for example when combined with routine indices such as CRP, NLR, or mGPS, rather than as a stand-alone prognostic biomarker.

Importantly, serum MMP8 did not correlate with intratumoral MMP8^+^ cell density, and dichotomized analyses using the prognostically informative cutoffs showed poor concordance between systemic and tissue MMP8 status. This argues against a simple spill-over explanation in which circulating MMP8 primarily reflects release from MMP8^+^ cells within the tumor. Rather, our data support partial uncoupling between systemic inflammatory activity and the local immune microenvironment in colorectal cancer. In line with this, intratumoral MMP8^+^ cell density associated with features of local immune contexture, including T cell infiltration, whereas systemic inflammatory markers (CRP, IL6) were not associated with intratumoral MMP8^+^ cell density. Together, these findings suggest that serum MMP8 and tissue MMP8 capture distinct biology, one reflecting systemic inflammatory state and the other reflecting local granulocyte infiltration within the tumor microenvironment.

Our findings contribute to the growing understanding of the dual roles of MMP8 in cancer biology. While MMP8 has been traditionally regarded as a pro-tumorigenic enzyme due to its matrix-degrading functions, emerging evidence supports its tumor-suppressive roles in certain contexts [36, 37]. For instance, studies have demonstrated that MMP8 can inhibit tumor invasion and metastasis by modulating inflammatory and immune responses [29, 38]. However, its systemic effects appear to be more closely aligned with tumor-promoting systemic inflammatory pathways [39]. This dichotomy mirrors findings observed for other MMP family members, such as MMP9, further emphasizing the complexity of their biological functions [40].

From a prognostic perspective, both serum MMP8 and intratumoral MMP8⁺ cell density showed only moderate discriminative ability in ROC analyses (AUC around 0.62–0.63), suggesting limited utility as stand-alone classifiers at the individual patient level. While low intratumoral MMP8^+^ density was independently associated with CRC-related death in multivariable models, serum MMP8 did not retain independent prognostic significance after adjustment for established clinicopathological factors. Independent validation is needed to determine whether tumor MMP8 provides incremental prognostic value beyond conventional markers and whether performance improves in defined patient subgroups.

This study has some limitations. First, serum sampling occurred within 0–30 days before surgery; because the exact sampling-to-surgery interval was not available for all patients, we could not assess or adjust for potential variability in serum MMP8 related to the timing of sampling. Second, the use of TMAs for MMP8^+^ cell and other immune cell density analyses restricts the assessment to limited regions of the tumor. Nevertheless, TMAs have been shown to reliably capture immune cell infiltration in previous studies [20]. Third, while we performed quantitative validation of the QuPath classifier against single-annotator cell-level ground truth, inter-observer agreement was not formally assessed because validation required manual labelling of > 100,000 cells. Future studies should evaluate inter-observer reproducibility using multi-annotator benchmarking or consensus labelling on a smaller subset of cores. Fourth, our study lacked information on postoperative cancer treatments. Future research should explore the role of MMP8 in relation to adjuvant therapies. Additionally, larger studies are required to assess the significance of MMP8 in specific patient subgroups, such as those with *BRAF*-mutated tumors. Investigating the functional interplay between tumor-localized and systemic MMP8 will further elucidate its diverse roles. The strengths of the study include a large and well-characterized study cohort, extensive clinicopathological and biomarker data, and the application of supervised machine-learning algorithms to enhance the precision and reproducibility of MMP8 immunohistochemistry analysis.

In summary, our study underscores the complex and context-dependent roles of MMP8 in CRC. While tumor-associated MMP8^+^ cells are linked to favorable outcome, serum MMP8 concentrations reflect systemic inflammation and are associated with poor outcomes in univariable analysis. These findings highlight the need for further research to unravel the multifaceted functions of MMP8 and to determine whether tissue MMP8^+^ cell density provides incremental prognostic value beyond established markers, and whether serum MMP8 has utility as a contextual marker of systemic inflammation in specific clinical settings.

## Supplementary Information

Below is the link to the electronic supplementary material.

Supplementary Material 1

## Data Availability

Data generated and/or analyzed during this study are not publicly available. The sharing of data will require approval from relevant ethics committees and/or biobanks. Further information including the procedures to obtain and access data of Finnish Biobanks are described at https://finbb.fi/en/fingenious-service.

## Abbreviations

AJCC: The American Joint Committee on Cancer
ASA: American Association of Anesthesiologists
AUC: area under the curve
CI: confidence interval
CRC: colorectal cancer
CRP: C-reactive protein
CSS: cancer-specific survival
CT: central tumor
DAB: 3,3’-Diaminobenzide
FFPE: formalin-fixed paraffin-embedded
HR: hazard ratio
mGPS: modified Glascow prognostic score
MSI: microsatellite instability
MMR: mismatch repair
MMP: matrix metalloproteinase
NLR: neutrophil-to-lymphocyte ratio
OS: overall survival
ROC: receiver operating characteristics
TMA: tissue microarray
