# Supplementary material for: Opposing prognostic roles of tumor-associated and circulating MMP8 in colorectal cancer
Source: Clin Exp Med. 2026 Apr 3;26(1):225. doi: 10.1007/s10238-026-02131-5 (PMC13199190; doi:10.1007/s10238-026-02131-5)
Supplement: Supplementary file 1 — Supplementary Material 1 [file 10238_2026_2131_MOESM1_ESM.pdf]

Table S1. Multivariable Cox regression models for cancer specific survival and overall survival according to tumor MMP8+ cell densities and serum MMP8 concentrations.

	Tumor MMP8+ cells		MMP8 serum concentration	
	CSS HR (95% CI)	OS HR (95% CI)	CSS HR (95% CI)	OS HR (95% CI)
Tumor MMP8+ cells			Not included	Not included
≤220/mm^2	1.76 (1.18-2.60)	1.19 (0.92-1.56)		
>220/mm^2	1 (referent)	1 (referent)		
MMP8 serum concentration	Not included	Not included		
≤100 ng/mL			1 (referent)	1 (referent)
>100 ng/mL			1.28 (0.83-1.95)	1.09 (0.77-1.54)
Age				
<65	1 (referent)	1 (referent)	1 (referent)	1 (referent)
65–75	1.65 (1.08-2.53)	1.66 (1.15-2.39)	1.32 (0.83-2.09)	1.54 (1.04-2.28)
>75	2.48 (1.59-3.85)	4.14 (2.93-5.85)	2.37 (1.50-3.75)	4.10 (2.85-5.91)
Sex				
Male	1 (referent)	1 (referent)	1 (referent)	1 (referent)
Female	1.02 (0.72-1.43)	0.80 (0.62-1.03)	0.96 (0.67-1.37)	0.85 (0.64-1.11)
Year of operation				
2006–2010	1 (referent)	1 (referent)	1 (referent)	1 (referent)
2011–2015	0.86 (0.58-1.30)	0.85 (0.63-1.15)	0.95 (0.63-1.45)	0.90 (0.66-1.24)
2016–2020	0.52 (0.34-0.81)	0.61 (0.44-0.85)	0.45 (0.28-0.74)	0.51 (0.35-0.74)
Tumor location				
Proximal colon	1 (referent)	1 (referent)	1 (referent)	1 (referent)
Distal colon	1.13 (0.74-1.74)	1.05 (0.75-1.47)	1.22 (0.76-1.95)	1.04 (0.73-1.49)
Rectum	0.97 (0.63-1.50)	1.03 (0.74-1.42)	1.10 (0.69-1.74)	1.07 (0.75-1.51)
Disease stage				
I–II	1 (referent)	1 (referent)	1 (referent)	1 (referent)
III	2.48 (1.48-4.15)	1.21 (0.88-1.67)	2.45 (1.41-4.24)	1.16 (0.82-1.64)
IV	17.6 (10.2-30.1)	7.59 (5.24-11.0)	16.6 (9.27-29.7)	7.37 (4.9-11.1)
Tumor grade				
Low-grade (well to moderately differentiated)	1 (referent)	1 (referent)	1 (referent)	1 (referent)
High-grade (poorly differentiated)	1.56 (1.01-2.41)	1.14 (0.80-1.63)	1.49 (0.95-2.36)	1.18 (0.80-1.72)
Lymphovascular invasion				
No	1 (referent)	1 (referent)	1 (referent)	1 (referent)
Yes	2.24 (1.42-3.53)	1.50 (1.12-2.01)	2.10 (1.29-3.40)	1.47 (1.07-2.03)
MMR status				
MMR proficient	1 (referent)	1 (referent)	1 (referent)	1 (referent)
MMR deficient	0.52 (0.25-1.09)	0.98 (0.63-1.52)	0.55 (0.25-1.23)	0.90 (0.56-1.47)
BRAF status				
Wild-type	1 (referent)	1 (referent)	1 (referent)	1 (referent)
Mutant	1.67 (0.94-2.98)	1.44 (0.95-2.20)	1.31 (0.68-2.54)	1.33 (0.84-2.11)

Abbreviations: CI, confidence interval; HR, hazard ratio; MMR, mismatch repair. The missing data in the serum MMP8 model (n=6 for BRAF status) were included in the majority category (BRAF wild-type) to limit the degrees of freedom.

Table S2. Univariable and multivariable Cox regression models for cancer-specific survival and overall survival according to serum MMP8 concentration (cut-off 56.6 ng/mL).

		Colorectal cancer-specific survival			Overall survival		
	No. of cases	No. of events	Univariable HR (95% CI)	Multivariable HR (95% CI)	No. of events	Univariable HR (95% CI)	Multivariable HR (95% CI)
Serum MMP8 concentration							
≤56.6 ng/mL	465	66	1 (referent)	1 (referent)	127	1 (referent)	1 (referent)
>56.6 ng/mL	205	63	2.25 (1.59-3.18)	1.28 (0.88-1.87)	96	1.69 (1.30-2.21)	1.02 (0.76-1.36)
P			<0.001	0.207		<0.001	0.893

Abbreviations: CI, confidence interval; HR, hazard ratio. Multivariable Cox proportional hazards regression models were adjusted for sex, age (<65, 65-75, >75), year of operation (2006–2010, 2011–2015, 2016-2020), tumor location (proximal colon, distal colon, rectum), disease stage (I–II, III, IV), tumor grade (well/moderately differentiated, poorly differentiated), lymphovascular invasion (negative, positive), mismatch repair (MMR) status (proficient, deficient), *BRAF* status (wild-type, mutant). The missing data in the serum MMP8 model (*n*=6 for *BRAF* status) were included in the majority category (*BRAF* wild-type) to limit the degrees of freedom.

Table S3. Baseline patient characteristics according to tumor MMP8⁺ cell density (cut-off 220/mm^2) and serum MMP8 concentration (cut-off 100 ng/mL) as binary variables.

		Tumor MMP8⁺ cells				Serum MMP8		
**Group**	**Total N**	Low	High	***P***	**N**	Low (%)	High (%)	***P***
**All cases**	760 (100%)	449 (59.1%)	311 (40.9%)		675 (100%)	583 (86.3%)	92 (13.6%)	
**Age**				0.748				>0.99
< 65	229 (30.1%)	133 (29.6%)	96 (30.9%)		210 (31.0%)	181 (31.0%)	29 (31.5%)	
≥ 65	531 (69.9%)	316 (70.4%)	215 (69.1%)		465 (69.0%)	402 (69.0%)	63 (68.5%)	
**Sex**				0.713				0.091
Male	402 (52.9%)	240 (53.5%)	162 (52.1%)		366 (54.2%)	324 (55.6%)	42 (45.7%)	
Female	358 (47.1%)	209 (46.5%)	149 (47.9%)		309 (45.8%)	259 (44.4%)	50 (54.3%)	
**Tumor location**				0.013				0.979
Proximal colon	319 (42.0%)	176 (39.2%)	143 (46.0%)		272 (40.3%)	234 (40.1%)	38 (41.3%)	
Distal colon	204 (26.8%)	138 (30.7%)	66 (21.2%)		183 (27.1%)	159 (27.3%)	24 (26.1%)	
Rectum	237 (31.2%)	135 (30.1%)	102 (32.8%)		220 (32.6%)	190 (32.6%)	30 (32.6%)	
**ASA**				0.952				0.642
1	39 (5.1%)	24 (5.8%)	15 (5.2%)		36 (5.8%)	32 (5.9%)	4 (5.5%)	
2	289 (38.0%)	169 (41.1%)	120 (41.2%)		259 (42.0%)	226 (41.5%)	33 (45.2%)	
3	313 (41.2%)	184 (44.8%)	129 (44.3%)		272 (44.1%)	244 (44.9%)	28 (38.4%)	
4	61 (8.0%)	34 (8.3%)	27 (9.3%)		50 (8.1%)	42 (7.7%)	8 (11.0%)	
**Depth of invasion**				<0.001				<0.001
T1	38 (5.0%)	15 (3.3%)	23 (7.4%)		47 (7.0%)	45 (7.7%)	2 (2.2%)	
T2	177 (23.3%)	85 (18.9%)	92 (29.6%)		154 (22.8%)	146 (25.0%)	8 (8.7%)	
T3	434 (57.1%)	275 (61.2%)	159 (51.1%)		384 (56.9%)	319 (54.7%)	65 (70.7%)	
T4	111 (14.6%)	74 (16.5%)	37 (11.9%)		90 (13.3%)	73 (12.5%)	17 (18.5%)	
**Nodal metastases**				<0.001				0.010
N0	440 (57.9%)	236 (52.6%)	204 (65.6%)		390 (57.8%)	350 (60.0%)	40 (43.5%)	
N1	192 (25.3%)	125 (27.8%)	67 (21.5%)		174 (25.8%)	141 (24.2%)	33 (35.9%)	
N2	128 (16.8%)	88 (19.6%)	40 (12.9%)		111 (16.4%)	92 (15.8%)	19 (20.7%)	
**Distant metastases**				0.009				<0.001
M0	676 (88.9%)	388 (86.4%)	288 (92.6%)		599 (88.7%)	536 (91.9%)	63 (68.5%)	
M1	84 (11.1%)	61 (13.6%)	23 (7.4%)		76 (11.3%)	47 (8.1%)	29 (31.5%)	
**TNM stage**				<0.001				<0.001
Stage I	174 (22.9%)	74 (16.5%)	100 (32.2%)		163 (24.1%)	157 (26.9%)	6 (6.5%)	
Stage II	252 (33.2%)	151 (33.6%)	101 (32.5%)		214 (31.7%)	186 (31.9%)	28 (30.4%)	
Stage III	250 (32.9%)	163 (36.3%)	87 (28.9%)		222 (32.9%)	193 (33.1%)	29 (31.5%)	
Stage IV	84 (11.1%)	61 (13.6%)	23 (7.4%)		76 (11.3%)	47 (8.1%)	29 (31.5%)	
**WHO grade**				0.345				0.015
Low	650 (85.5%)	389 (86.6%)	261 (83.9%)		579 (85.8%)	508 (87.1%)	71 (77.2%)	
High	110 (14.5%)	60 (13.4%)	50 (16.1%)		96 (14.2%)	75 (12.9%)	21 (22.8%)	
**Lymphovascular invasion**				0.001				<0.001
No	415 (54.6%)	223 (49.7%)	192 (61.7%)		376 (55.7%)	341 (58.5%)	35 (38.0%)	
Yes	345 (45.4%)	226 (50.3%)	119 (38.3%)		299 (44.3%)	242 (41.5%)	57 (62.0%)	
***BRAF* status**				0.020				0.739
Wild-type	653 (85.9%)	397 (88.4%)	256 (82.3%)		587 (87.9%)	508 (87.1%)	79 (85.9%)	
Mutant	107 (14.1%)	52 (11.6%)	55 (17.7%)		88 (13.0%)	75 (12.9%)	13 (14.1%)	
**MMR status**				<0.001				0.439
MMR-proficient	638 (83.9%)	405 (90.2%)	233 (74.9%)		105 (15.6%)	88 (15.1%)	17 (18.5%)	
MMR-deficient	122 (16.1%)	44 (9.8%)	78 (25.1%)		570 (84.4%)	495 (84.9%)	75 (81.5%)	
**mGPS**				0.922				<0.001
mGPS0	555 (78.6%)	328 (79.0%)	227 (78.0%)		527 (79.2%)	480 (83.2%)	47 (53.5%)	
mGPS1	96 (13.6%)	56 (13.5%)	40 (13.7%)		92 (13.8%)	64 (11.1%)	28 (31.8%)	
mGPS2	55 (7.8%)	31 (7.5%)	24 (8.2%)		46 (6.9%)	33 (5.7%)	13 (14.8%)	

Table S4. Tumor MMP8⁺ cell density in relation to serum MMP8 concentration.

	N (%)	MMP8$^+$ cell density		*P* value
		Low (≤220 cells/mm^2)	High (>220 cells/mm^2)	
Serum MMP8 level				0.563
Low (≤100 ng/mL)	571 (86.5%)	340 (85.9%)	231 (87.5%)	
High (>100 ng/mL)	89 (13.5%)	56 (14.1%)	33 (12.5%)	

P value was calculated using the Fisher's exact test.

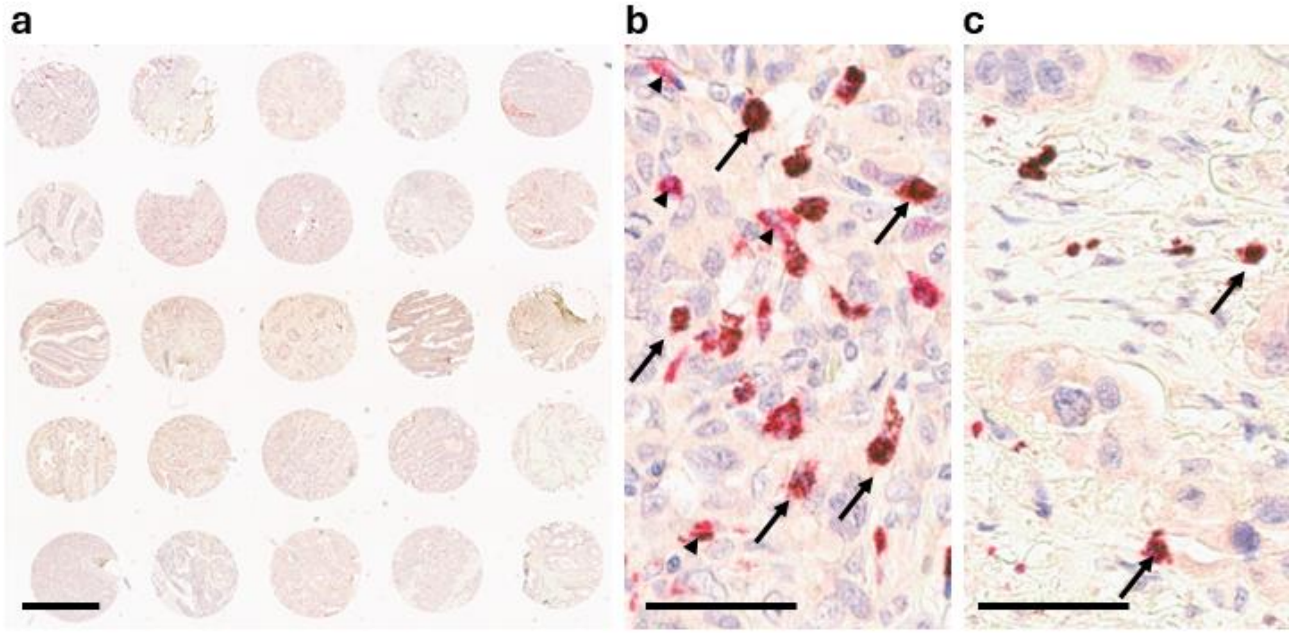

Figure S1. MMP8/CEACAM8 double immunohistochemistry on 25 tumours. MMP8 is indicated by a brown chromogen and CEACAM8 by a red chromogen. **(a)** An overview of the tissue microarray slide. Scale bar indicates 1 mm. **(b-c)** Two close-up views showing examples of MMP8+CEACAM8+ cells (arrows) and MMP8-CEACAM8+ cells (arrowheads). Scale bars indicate 50 μm.

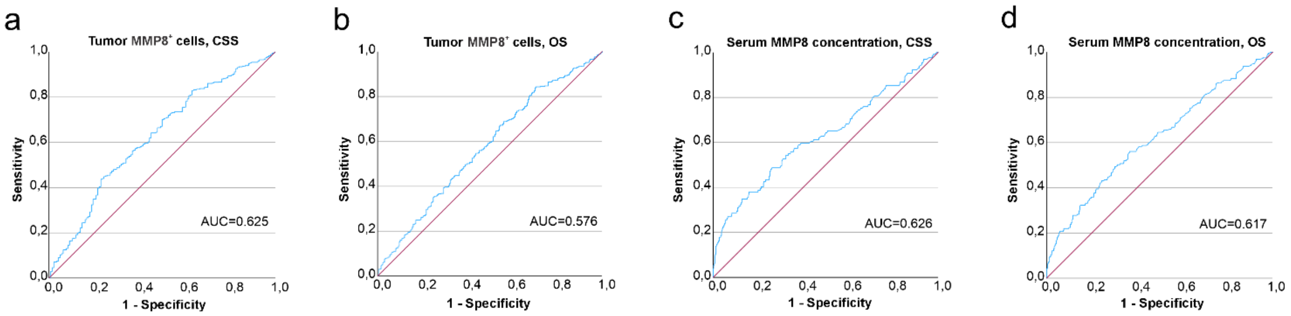

Figure S2. Receiver operating characteristics (ROC) analysis. (a-b) ROC curves for tumor MMP8+ cell densities for **(a)** cancer-specific survival (CSS) and **(b)** overall survival (OS). **(c-d)** ROC curves for serum MMP8 concentrations **(c)** CSS and **(d)** OS.
